# International Movement of Livestock and Lack of Regulation for Internal Parasites Monitoring

**Published:** 2019

**Authors:** Alireza SAZMAND

**Affiliations:** Department of Pathobiology, Faculty of Veterinary Science, Bu-Ali Sina University, Hamedan, Iran

## Dear Editor-in-Chief

As a parasitology researcher, I do have serious concerns about establishment of new and emerging parasitic infections by recent massive import of live ruminants to the country.

Illegal export of live ruminants from Iran to neighboring countries due to abnormal foreign currency exchange rates has made official import of live small ruminants from foreign countries unavoidable in order to balance the meat price in the market. According to the State Livestock Affairs Logistics 50,000 livestock will be imported weekly, and importation of live animals is planned to be continued in coming months until normalization of the prices in the market (1). Reading the news on Friday 15th of February 2019 I got interested to check if there are quarantine and biosecurity regulations regarding parasitic diseases in Iran.

According to the regulations of the Iranian Veterinary Organization, ruminants should not show signs or symptoms of certain viral (foot-and-mouth disease, peste des petits ruminants, rinderpest, bluetongue, scrapie, rabies, pox, vesicular stomatitis) and bacterial (anthrax, brucellosis, tuberculosis, contagious bovine pleuropneumonia and mycoplasma mastitis) diseases on the day of shipment. In addition, the livestock have to be originated from establishments with no case of these diseases for twenty days up to 12 months preceding export (depending on the pathogen) and/or have to be vaccinated at least twenty days (but not more than 6 months) prior to import. Also, animals intended for import to Iran have to be free from all wounds, external parasitic infestations and myiasis, and should be kept for a minimum of 21 days pre-import (2,3). However, in my searches among official documents I found no regulation for checking helminthic infections in herbivorous animals intended for import into Iran.

International animal and meat trade plays a very important role in spread of infectious diseases including zoonoses (4–6). For the year 2019, World Organization for Animal Health (OIE) established a single list of 117 diseases, infections and infestations “giving all listed diseases the same degree of importance in international trade” instead of the former Lists A and B to be “in line with the terminology of the Sanitary and Phytosanitary Agreement of the World Trade Organization” (WTO). In the new list, diseases, infections and infestations of cattle, sheep and goats with *Echinococcus granulosus*, *E. multilocularis*, *Trichinella* spp., New World screwworm (*Cochliomyia hominivorax*), Old World screwworm (*Chrysomya bezziana*), Surra (*Trypanosoma evansi*), babesiosis, theileriosis, trichomonosis and trypanosomosis (tsetse-transmitted) have been declared as hazardous (7). Currently, none of these diseases except screwworms are being checked in imported livestock to Iran.

In general, policy-makers have mainly focused on contagious viral and bacterial diseases of livestock with high morbidity and mortality rates while introduction and establishment of new and emerging parasitic infections has been neglected. Although not listed by OIE and not obligatory by World Trade Organization, livestock infections by vector-borne parasites including filaroid nematodes such as *Thelazia*, *Setaria* and *Onchocerca* as well as *Besnoitia* with a wide range of insect vectors and geographical distribution in the world are among the transmissible diseases important to keep eyes on. Moreover, selective treatment of internal parasites in ruminants in country of origin will help to prevent entrance of helminthes to our country barns, flocks and pastures. I wish Iranian Veterinary Organization will act as pioneer of appropriate regulations in international trade.

Currently, the interval between import of live ruminants to the country and slaughter is short, and movements of animals are controlled, so that risk of the establishment of parasitic infections is low. However, as a veterinary parasitology researcher I suggest the inclusion of parasitic infections in the list of transmissible diseases under mandatory monitoring prior to import into Iran.
